# Deviation of eyes and head in acute cerebral stroke

**DOI:** 10.1186/1471-2377-6-23

**Published:** 2006-06-26

**Authors:** M Fruhmann Berger, RD Proß, UJ Ilg, H-O Karnath

**Affiliations:** 1 Section Neuropsychology, Center of Neurology, Hertie-Institute for Clinical Brain Research, University of Tübingen, Tübingen, Germany; 2 Oculomotor Laboratory, Hertie-Institute for Clinical Brain Research, University of Tübingen, Tübingen, Germany

## Abstract

**Background:**

It is a well-known phenomenon that some patients with acute left or right hemisphere stroke show a deviation of the eyes (Prévost's sign) and head to one side. Here we investigated whether both right- and left-sided brain lesions may cause this deviation. Moreover, we studied the relationship between this phenomenon and spatial neglect. In contrast to previous studies, we determined not only the discrete presence or absence of eye deviation with the naked eye through clinical inspection, but actually measured the extent of horizontal eye-in-head and head-on-trunk deviation. In further contrast, measurements were performed early after stroke onset (1.5 days on average).

**Methods:**

Eye-in-head and head-on-trunk positions were measured at the bedside in 33 patients with acute unilateral left or right cerebral stroke consecutively admitted to our stroke unit.

**Results:**

Each single patient with spatial neglect and right hemisphere lesion showed a marked deviation of the eyes and the head to the ipsilesional, right side. The average spontaneous gaze position in this group was 46° right, while it was close to the saggital body midline (0°) in the groups with acute left- or right-sided stroke but no spatial neglect as well as in healthy subjects.

**Conclusion:**

A marked horizontal eye and head deviation observed ~1.5 days post-stroke is not a symptom associated with acute cerebral lesions per se, nor is a general symptom of right hemisphere lesions, but rather is specific for stroke patients with spatial neglect. The evaluation of the patient's horizontal eye and head position thus could serve as a brief and easy way helping to diagnose spatial neglect, in addition to the traditional paper-and-pencil tests.

## Background

The spontaneous horizontal deviation of the eyes is a striking symptom in acute stroke. Correspondingly, its evaluation is part of different clinical stroke scales, including the National Institutes of Health Stroke Scale [1], the European Stroke Scale [2], or the Scandinavian Stroke Scale [3,4]. It seems as if this sign occurs after both right-sided as well as left-sided stroke, steering the eyes towards the respective lesion side. Already in 1865 Jean Louis Prévost reported that "in all cases [of acute hemiplegia] I have observed, the two ocular axes were always deviated to the side opposite the paralysis, thus the two eyes looked towards the damaged hemisphere" [[5], p. 649]. Since then, only few studies have been carried out investigating this symptom in patients with unilateral cerebral stroke.

From these studies, five have particularly addressed the relationship between a deviation of the eyes (and of the head) and spatial neglect [6-10]. However, none of these studies could sufficiently answer the question whether or not a spontaneous ipsilesional deviation of the eyes and the head is *specific*for spatial neglect. Ringman and coworkers [10] showed that horizontal eye deviation predominantly occurs after right hemisphere lesions. However, the authors determined the frequency of eye deviation only in patients with neglect but did not report how many patients without neglect showed the same behaviour. De Renzi and co-workers [6] investigated spatial neglect with a delay of 14–18 days after stroke. The relation between eye/head deviation and neglect therefore remained open for those patients who did already recover from eye/head deviation at that time. In contrast, the study by Tijssen [7] claimed to examine eye deviation and spatial neglect at the first day after admission. However, like Ringman et al. [10], the examination included none of the traditional tests used to diagnose spatial neglect. Tijssen [7] investigated visual and tactile extinction, asomatognosia, and anosognosia; Ringman et al. [10] tested patients by simultaneous tactile stimulation (tactile extinction) and by a verbal description of a complex picture scene (Cooky Theft Picture, [11]). Like De Renzi et al. [6] and Tijssen [7], also Kömpf and Gmeiner [8] did not systematically investigate patients *without*eye/head deviation for spatial neglect.

Most importantly, these studies determined the discrete presence or absence of eye deviation with the naked eye through clinical inspection. Only one of the previous studies actually measured eye and head position, which allowed for a quantitative analysis of the patients' behaviour [9]. The authors found a close relation between a marked rightward deviation of the eyes and the head and the diagnosis of spatial neglect. However, as in the studies of De Renzi et al. [6] and of Tijssen [7], no information was obtained concerning the patients' neglect behaviour in the very acute stage of the stroke, i.e. early after admission. Moreover, like Kömpf and Gmeiner [8], Fruhmann Berger and Karnath [9] did not investigate patients with left hemisphere stroke.

Thus, it still remains open whether the spontaneous deviation of the eyes and the head is a sign of spatial neglect and/or right hemisphere lesion, or whether it also occurs regularly after left hemisphere stroke. The present study aimed to answer this question. In order to avoid the methodological limitations of the previous studies, we investigated 33 patients with acute unilateral left or right hemisphere first-ever stroke as soon as possible after admission to our stroke unit by recording eye and head position directly at the bedside.

## Methods

### Subjects

The aim of the study was to measure stroke patients' spontaneous eye-in-head and head-on-trunk positions as early as possible after stroke onset. Between the day of stroke and the following three days (i.e. days 0 to 3) – dependent on the patient's general constitution and the need of medical attendance in this very acute stage of the stroke – we investigated a sample of 33 patients with unilateral first-ever stroke verified by magnetic resonance imaging (MRI) and/or computed tomography (Spiral-CT). Patients with diffuse or bilateral brain lesions or with lesions restricted to the brainstem or cerebellum were excluded. The sample consisted of 8 patients with acute right-hemisphere lesions and spatial neglect (RBD+), 9 patients with right-hemisphere lesions without spatial neglect (RBD-), and 16 patients with left-hemisphere stroke without spatial neglect (LBD-). In addition, 15 healthy subjects (NBD) without brain injury were investigated. During the time period of the present investigation no patient with spatial neglect following a left-sided stroke could be investigated. All subjects gave their informed consent to participate in the study, which was performed in accordance with the ethical standards laid down in the 1964 Declaration of Helsinki and was approved by the local ethics committee (Ethik-Kommission der Medizinischen Fakultät, Eberhards-Karls-Universität, Tübingen, Germany). Clinical, demographic, and anatomical data are presented in Table 1.

### Clinical examination

The clinical and the experimental investigations were carried out in one session or (if not possible) at least at the same day. The level of consciousness of each patient was determined using the Glasgow Coma Scale [12]. Visual field defects were assessed by the common neurological confrontation examination. Severity of paresis was scored with the usual clinical ordinal scale, where '0' represents no trace of movement and '5' normal movement. Aphasic symptoms were investigated by spontaneous speech, picture naming, and auditory comprehension of single words and whole sentences. None of the subjects had a history of vestibular or oculomotor abnormalities.

Spatial neglect was diagnosed when patients fulfilled the criterion in at least two of the following traditional paper-and-pencil tests: the "Letter cancellation" task [13], the "Bells test" [14], the "Albert's test" [15], or a copying task [16]. In the letter cancellation test, a horizontally oriented 21 × 29.7 cm sheet of paper was presented on which 60 target letters 'A' are distributed amid distractors, 30 on the right half of the page and 30 on the left. Patients were asked to cancel all of the targets. They were classified as suffering from spatial neglect when omitting more than four contralateral located targets. The Bells test consists of seven columns each containing five targets (bells) amid 40 distractors. Three of the seven columns (= 15 targets) are on the left side of a horizontally oriented 21 × 29.7 cm sheet of paper, one is in the middle, and three are on the right side (= 15 targets). Patients were asked to cancel all of the targets. More than five contralateral located target omissions were taken to indicate neglect. The Albert's test consists of seven columns of black lines. Three of the seven columns (= 12 targets) are on the left side of a horizontally orientated 21 × 29.7 cm sheet of paper, one column, containing 5 lines, is in the middle, and three columns (= 12 targets) are on the right side. Patients again had to cancel all targets. More than one contralateral located target omission was taken to indicate neglect. In the copying task, patients were asked to copy a complex multi-object scene consisting of four figures (a fence, a car, a house, and a tree), two in each half of a horizontally oriented 21 × 29.7 cm sheet of paper. Omission of at least one of the contralateral features of each figure was scored as 1, and omission of each whole figure was scored as 2. One additional point was given when contralateral located figures were drawn on the ipsilesional side of the paper sheet. The maximum score was 8. A score higher than 1 (i.e. > 12.5% omissions) was taken to indicate neglect.

### Apparatus

In order to measure the patient's spontaneous orientation of eye-in-head and head-on-trunk in the acute stage after stroke (0 to 3 days after stroke onset), the investigation had to take place directly on the stroke unit at the bedside. The spontaneous horizontal eye-in-head position was measured by electrooculography (EOG; [17,18]). We applied three silver/silver chlorine (Ag/AgCl) electrodes, two at the outer canthus of each eye and one at the patient's forehead, the latter serving as the reference electrode. All signals passed a lowpass filter (20–30 Hz) before they were amplified by a conventional DC amplifier. The sample rate was 70 Hz. For calibrating the EOG, the patient was asked to look on light emitting diodes (LEDs) displayed on a black cardboard, presented 30 cm in front of the patient's eye level. The LEDs were presented at 0°, +/-10°, and +/-20° of visual angle with respect to the patient's mid-sagittal head axis without modifying the patient's spontaneously chosen head position.

The spontaneous horizontal head-on-trunk position was measured by a standard orthopaedic graphometer circle. This tool consists of two measuring tubes. One tube was aligned parallel to the coronal plane defined by the patient's left and right acromion; the other was oriented along the line between the nasion and inion. The resulting head-on-trunk angle was measured and its duration marked on-line in the EOG data file. Since the patients were asked to rest in a comfortable position (see below), part of them did not move the head at all during data recording. This resulted in a single head position value for the entire recording period. When a patient changed the head position, the new head-on-trunk angle was determined and its duration again marked on-line in the EOG data file. The measured head-on-trunk angles were weighted according to their relative portion of the overall acquisition time and the mean head-on-trunk position was calculated.

Horizontal gaze orientation was calculated by adding the mean eye-in-head and head-on-trunk angles of each patient. Horizontal head-on-trunk and gaze co-ordinate 0° was defined by the subject's mid-sagittal body axis. Eye-in-head co-ordinates were head-centred, i.e. co-ordinate 0° was aligned with the head's mid-sagittal axis. Positive values thus indicated locations right of these centres, negative values locations on the left.

### Procedure

The investigation took place on the stroke unit. Under normal daylight conditions, the patient was seated in an upright position in either the sickbed or the wheelchair. Close-drawn white curtains separating the single sickbeds on their left, right, and frontal sides provided a balanced visual environment. After calibration of the EOG, the patient was asked to rest in a comfortable, relaxed position with eyes open and without talking. Before data recording started, experimenters were positioned out of the patient's sight, right behind the sickbed or the wheelchair to avoid any disturbances in the visual fields. The patient was informed that data recording now will start and that they should keep their comfortable position and – if possible – not move too much until data recording is finished. No further communication took place during the following period of data acquisition.

Data were recorded for 90s and were stored on hard disc for off-line analysis. Any disturbances during data acquisition, e.g. when patients closed the eyes, started to talk, or moved the trunk were marked on-line in the data file. These periods were excluded from data analyses. After terminating data recording, calibration of the EOG was repeated. Two circles of data acquisition were carried out, summing up for a total recording period of 180s per patient.

## Results

**Figure 1:**
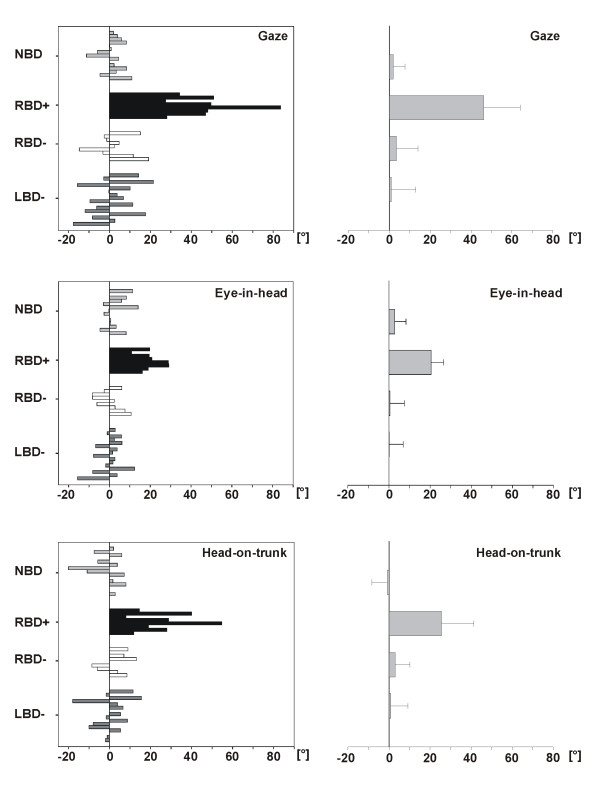
**Left panel: **Individual horizontal position (in degrees of visual angle) of gaze, eye-in-head, and head-on-trunk in all 48 subjects, each averaged over the 180s period of data acquisition. NBD, non brain-damaged healthy subjects (*light grey bars*); RBD+, right hemisphere stroke patients with spatial neglect (*black bars*); RBD-, right hemisphere stroke patients without spatial neglect (*white bars*); LBD- left hemisphere stroke patients without spatial neglect (*dark grey bars*). During the time period of the present investigation no patient with spatial neglect following left hemisphere stroke could be investigated. **Right panel: **Mean horizontal position (and standard deviation) of the different groups and variables.

Figure 1 gives the individual horizontal gaze, eye-in-head, and head-on-trunk positions for all 48 subjects, each averaged over the 180 s period of data acquisition. We found a huge deviation of all three parameters in the group of neglect patients (RBD+) compared to all other groups without spatial neglect (RBD-, LBD-, NBD). Moreover, we found this deviation of gaze, eye-in-head, and head-on-trunk position in every single patient with spatial neglect (RBD+) and exclusively (= 100%) towards the ipsilesional, right side. In contrast, the smaller deviations of gaze, eye-in-head, and head-on-trunk in the RBD-, LBD-, and NBD group were balanced in direction towards the left and the right side (cf. Fig. 1). For statistical comparison of the four groups (RBD+, RBD-, LBD-, NBD), we conducted separate one-way ANOVAs for gaze, eye-in-head, and head-on-trunk position, followed by post-hoc tests using a Bonferroni adjusted significance level of α = 0.05.

### Gaze position

We found a highly significant difference between the four groups (*F*_3 _= 33.12, *p*< 0.001). Post-hoc comparisons revealed that this effect was due to the marked difference between the neglect patients' mean horizontal gaze position of 46.1° (SD 18.0°) compared to each other group, showing comparable spontaneous gaze positions close to the mid-sagittal body axis. The right hemisphere stroke patients without neglect had an average gaze position of 3.5° (SD 10.5°; *t*_15 _= 6.06, *p*< 0.001), the left hemisphere stroke patients without neglect a mean of 1.0° (SD 12.0°; *t*_22 _= 7.35, *p*< 0.001), and the healthy control subjects a mean of 1.9° (SD 5.8°; *t*_8 _= 6.77, *p*< 0.001).

### Eye-in-head position

The one-way ANOVA conducted for eye-in-head position revealed a significant main effect of factor "group" (*F*_3 _= 20.81, *p*< 0.001). Subsequent post-hoc comparisons showed that the neglect patients' average spontaneous eye-in-head position was markedly deviated to the right side compared to each other group (RBD-: *t*_15 _= 6.21, *p*< 0.001, LBD-: *t*_22 _= 7.12, *p*< 0.001; NBD: *t*_21 _= 7.07, *p*< 0.001). The mean horizontal eye-in-head position of the neglect group deviated 20.5° (SD 6.1°) to the ipsilesional side, while the averaged positions of the RBD-(mean = 0.5°, SD 7.1°), LBD- (mean = 0.1°, SD 6.9°), and NBD (mean = 2.8°, SD 5.5°) group were close to the head midline.

### Head-on-trunk position

The analysis of head-on-trunk position also obtained a significant difference between the four groups (*F*_3 _= 15.53, *p*< 0.001). Again this effect was due to a marked difference between the group of neglect patients and all other groups. The neglect patients' mean horizontal head-on-trunk position showed a substantial deviation of 25.6° (SD 15.8°) to the ipsilesional, right side. In contrast, the mean positions of the right hemispheric stroke patients without neglect (RBD-: mean = 3.0°, SD 7.2°; *t*_15 _= 3.88, *p*= 0.001), the left hemispheric stroke patients without neglect (LBD-: mean = 0.9°, SD 8.4°; *t*_9 _= 4.15, *p*= 0.002), and the healthy control subjects (NBD: mean = -0.9°, SD 7.5°; *t*_9 _= 4.49, *p*= 0.002) were close to the mid-sagittal body axis.

## Discussion

The present study investigated the relationship between the spontaneous horizontal eye (Prévost's sign) and head deviation and spatial neglect in patients with acute left- or right-sided cerebral stroke. Our aim was to measure eye-in-head and head-on-trunk positions as early as possible after stroke. We were able to investigate our patients on average 1.5 days after the onset of neurological symptoms. At this time point we observed that each single patient with spatial neglect and a right-sided lesion showed a marked spontaneous deviation of the eyes and the head towards the ipsilesional, right side. The average deviation of the spontaneous horizontal gaze position in the neglect group was enormous with 46° towards the right. Such marked deviation of the eyes and the head was neither observed in left nor in right hemisphere stroke patients without spatial neglect nor in healthy subjects. Spontaneous horizontal eye-in-head and head-on-trunk positions in these latter groups varied around the sagittal trunk midline (0°), leading to an average position very close to this axis.

Our results do not allow to draw conclusions for the time period between 0 and (on average) 1.5 days after the onset of neurological symptoms. It may be possible that in this short time period after stroke onset marked eye and head deviation also occurs with left hemisphere lesions. If this indeed would be true, the present results would indicate that such deviation after left-sided cerebral stroke recovers extremely fast (within 1.5 days on average), while the eye and head deviation associated with right-sided lesions remains. However, investigation of this question must remain the issue of future studies.

It is known that spatial neglect occurs asymmetrically after right hemisphere stroke, as e.g. aphasia is observed asymmetrically after left hemisphere lesions [19-21]. Consistent with this notion, we did not observe spatial neglect caused by left hemisphere lesion among the 33 admitted stroke patients of the present study. However, as patients can show aphasia after right hemisphere stroke, we expect to find subjects with spatial neglect after left hemisphere lesion in other samples or samples of larger size. Based on the present findings, we predict that such neglect patients show a spontaneous horizontal eye-in-head and head-on-trunk deviation towards the left side, comparable to the marked rightward deviation observed in each of the neglect patients of the present study.

Prévost [5] pointed out that the deviation of the eyes and the head "can be an invaluable indicator for the [stroke] diagnosis" (p. 649). While Prévost assumed that the deviation of eyes and head occurred symmetrically after both left and right hemisphere lesion, the present study clearly showed that a marked horizontal deviation of eyes and head observed ~1.5 days post-stroke is tightly connected with spatial neglect. The same is true at later stroke stages [9]. Prévost's sign and spatial neglect thus seem to reflect the same phenomenon, namely a constantly biased orienting towards the right side. The present data show that such a bias occurs predominantly after right-sided stroke, indicating that the underlying function leading to such a bias is represented asymmetrically in the human hemispheres.

What is the consequence of the neglect patients' marked spontaneous eye and head deviation to the right side? We know that patients with spatial neglect carry out exploratory movements predominantly on the ipsilesional side when searching for targets, reading, copying, etc. [22-25]. It seems as if the spontaneous deviation of the eyes and the head provokes this asymmetric behaviour. Neglect patients appear to carry out visual and tactile movements around the deviated centre of eye and head position, leading to neglect of information on the contralesional, left side.

The present results may have implications for acute stroke diagnosis. As acute language disorders strongly argue for a stroke in the left hemisphere, our data suggest that a marked horizontal eye and head deviation observed ~1.5 days post-stroke is a clear sign for spatial neglect (and typically right hemisphere stroke in those patients). In addition to the traditional paper-and-pencil tests, the evaluation of stroke patient's horizontal eye and head position thus could serve as a brief and easy way helping to diagnose the disorder.

The marked deviation of eyes and head towards the ipsilesional side in neglect patients could point to a close relationship of spatial neglect to asymmetric function of the multisensory (vestibular) system [26]. Unilateral vestibular loss in neurological patients or asymmetrical stimulation of one vestibular organ in healthy subjects provoke a shift of the average horizontal position of the eyes and the head towards the affected side [27-29]. Such a bias of eyes and head towards the right likewise is observed in stroke patients with spatial neglect.

## Conclusion

Our present data allow to conclude that a marked spontaneous horizontal deviation of the eyes and the head observed ~1.5 days post-stroke is not a symptom associated with acute cerebral lesions per se, nor is a general symptom of right hemisphere lesion, but rather is a specific sign of spatial neglect. Our results necessitate to modify Prévost's [5] original assumption that eye and head deviation occurs symmetrically with both left- and right-sided stroke.

## Competing interests

The author(s) declare that they have no competing interests.

## Authors' contributions

M.F.B. and H.-O.K. conceived the experiment and drafted the manuscript. M.F.B. and R.D.P. carried out data acquisition and analyses. U.J.I. programmed the experimental set-up. All authors read and approved the final manuscript.

## Pre-publication history

The pre-publication history for this paper can be accessed here:



## Figures and Tables

**Table 1:** Demographic and clinical data of the 33 patients with and without spatial neglect and of the 15 non-brain-damaged subjects.

			**Neglect**	**No neglect**	**No neglect**	**No neglect**
			Neg	RBD	LBD	NBD
Number			8	9	16	15
Sex			4 f, 4 m	3 f, 6 m	6 f, 10 m	11 f, 4 m
Age (yr)		Mean (SD)	57.6 (14.6)	62.3 (10.3)	64.7 (9.9)	59.9 (11.8)
Etiology		Infarct	8	6	11	
		Haemorrhage	0	3	5	
Time since lesion (d)		Mean (SD)	1.4 (0.9)	1.4 (0.9)	1.7 (0.9)	
Glasgow Coma Scale		Mean (SD)	4.9 (1.0)	5.0 (0.9)	4.7 (1.1)	
Visual field defect		% present	12.5	22.2	12.5	
Contralateral paresis		% present	100	55.6	56.3	
Aphasia		% present	0	0	87.5	
Neglect		% present	100	0	0	
Lesion location			NEG1 Bg	RBD1 Bg	LBD1 Th	
			NEG2 F, T, I, Bg	RBD2 P, T	LBD2 T, O	
			NEG3 F, P, T, O, I	RBD3 T, I, Bg	LBD3 F, I, Bg	
			NEG4 Bg	RBD4 O	LBD4 Bg	
			NEG5 F, T, I	RBD5 Bg	LBD5 Bg	
			NEG6 P	RBD6 Bg	LBD6 F, I, Bg	
			NEG7 T	RBD7 Bg	LBD7 Th	
			NEG8 F, T, I, Bg	RBD8 Bg	LBD8 P, T, I	
				RBD9 Bg, Th	LBD9 P, T, I	
					LBD10 P	
					LBD11 O	
					LBD12 I, Bg	
					LBD13 I, Th, Bg	
					LBD14 Th	
					LBD15 T	
					LBD16 Swm	
Letter cancellation	Left	Mean (SD)	8.5 (10.6)	29.3 (0.7)	27.3 (2.1)	
	Right	Mean (SD)	20.3 (8.0)	29.6 (0.5)	27.8 (1.8)	
Bells test	Left	Mean (SD)	4.0 (4.1)	13.9 (1.1)	13.8 (1.4)	
	Right	Mean (SD)	11.2 (4.1)	14.6 (0.7)	13.3 (2.1)	
Albert's test	Left	Mean (SD)	8.4 (9.0)	18.0 (0)	18.0 (0)	
	Right	Mean (SD)	14.0 (6.4)	18.0 (0)	17.8 (0.8)	
Copying (% omitted)		Mean (SD)	41.7 (34.2)	1.4 (4.2)	4.5 (7.9)	

NEG, right brain damaged stroke patients with spatial neglect; RBD, right brain damaged stroke patients without spatial neglect; LBD, left brain damaged stroke patients without spatial neglect; NBD, non-brain-damaged subjects; f, female; m, male; F, frontal; P, parietal; T, temporal; O, occipital; I, insula; Th, thalamus; Bg, basal ganglia; Swm, subcortical white matter.
