# Correction: deviation of eyes and head in acute cerebral stroke

**DOI:** 10.1186/1471-2377-6-49

**Published:** 2006-12-28

**Authors:** M Fruhmann Berger, RD Proß, UJ Ilg, H-O Karnath

**Affiliations:** 1 Section Neuropsychology, Center of Neurology, Hertie-Institute for Clinical Brain Research, University of Tübingen, Tübingen, Germany; 2 Oculomotor Laboratory, Hertie-Institute for Clinical Brain Research, University of Tübingen, Tübingen, Germany

## Abstract

This is a correction article

## Correction

Much to our regret we have to inform you about a mistake/misprint in our recent article: Fruhmann Berger et al [1].

In Table 1, the mean scores of the Glasgow Coma Scale reported for the stroke patient groups should read:

Neg 14.6 (0.5);

RBD 14.9 (0.3);

LBD 14.2 (1.1).

## Pre-publication history

The pre-publication history for this paper can be accessed here:



## References

[B1] Fruhmann Berger M, Proß RD, Ilg UJ, Karnath H-O (2006). Deviation of eyes and head in acute cerebral stroke. BMC Neurology.

